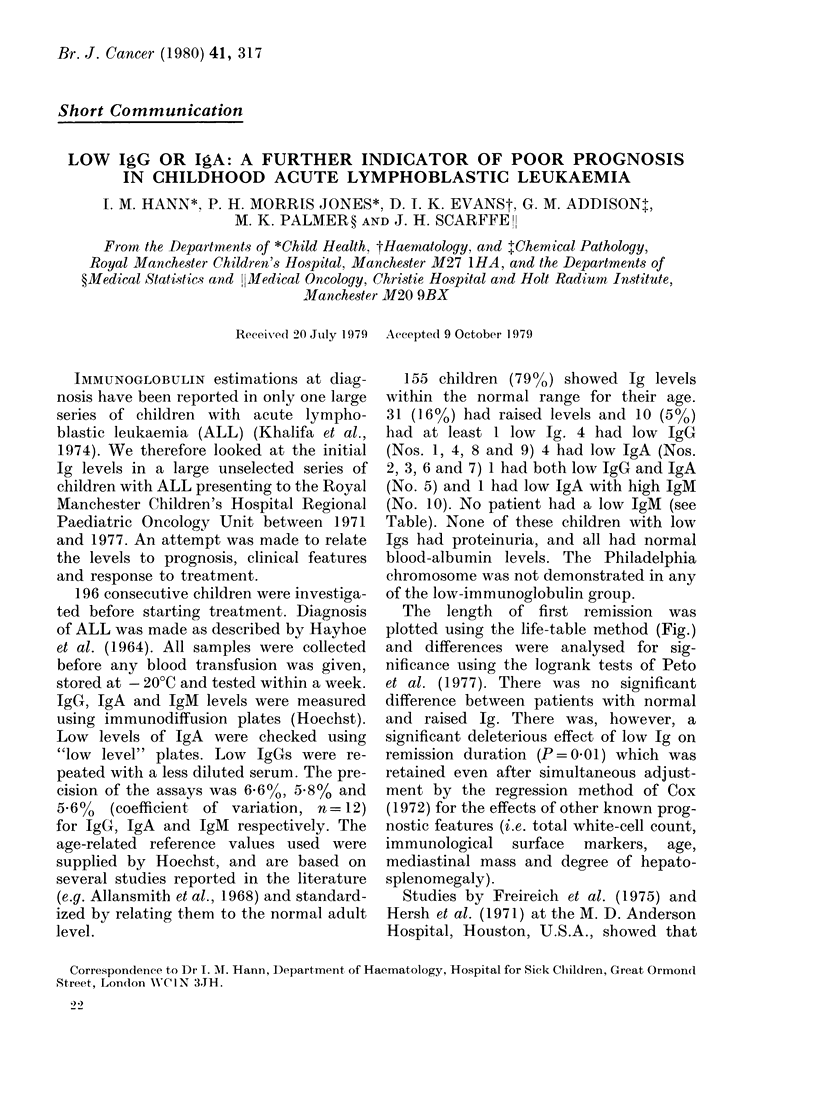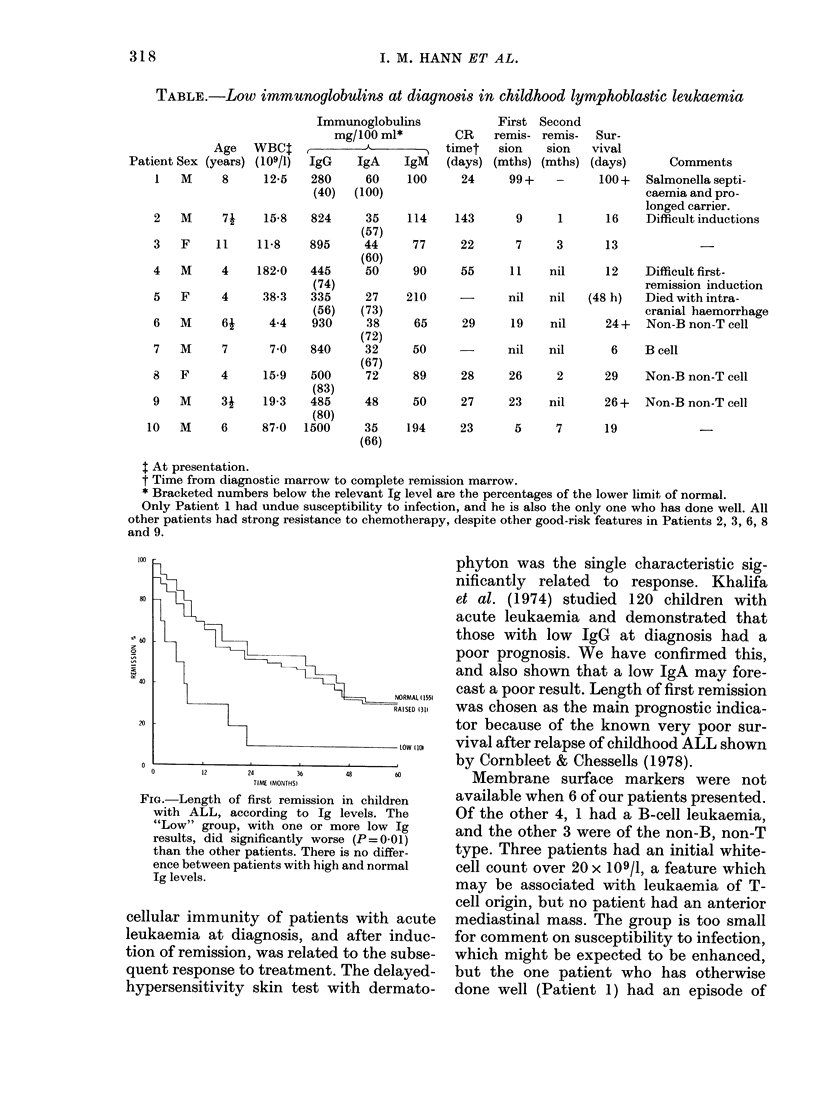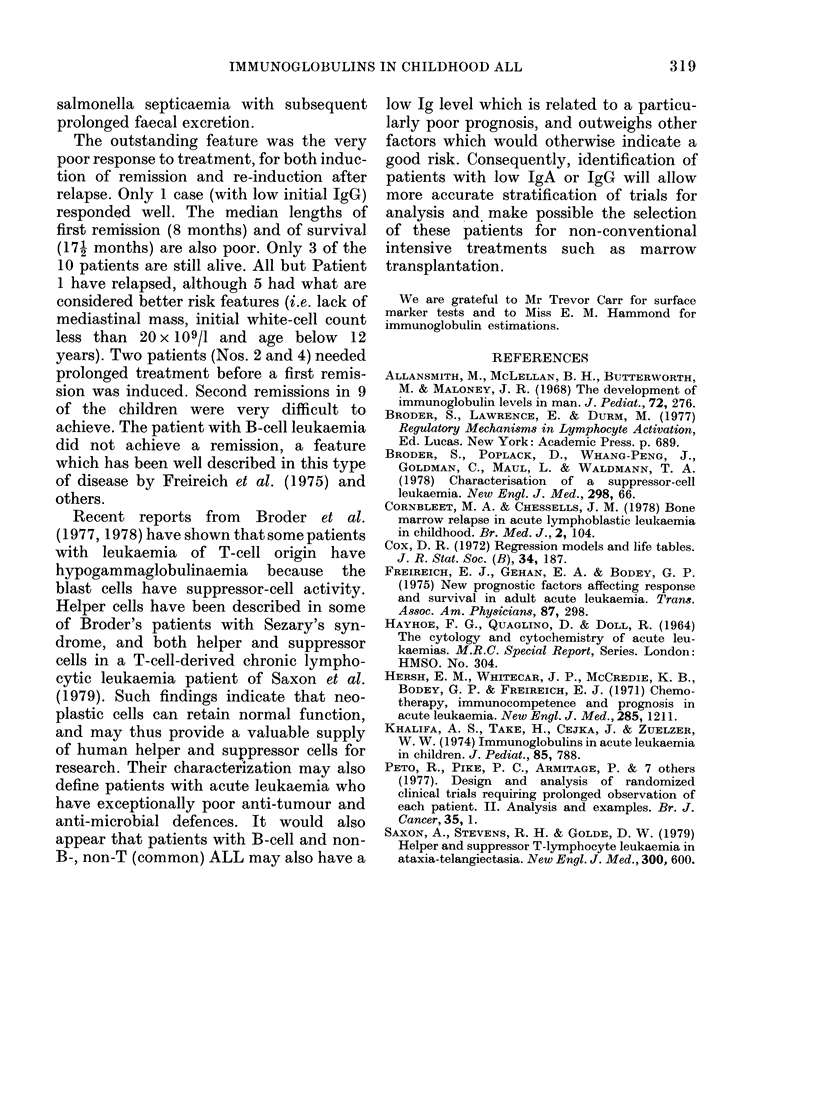# Low IgG or IgA: a further indicator of poor prognosis in childhood acute lymphoblastic leukaemia.

**DOI:** 10.1038/bjc.1980.47

**Published:** 1980-02

**Authors:** I. M. Hann, P. H. Jones, D. I. Evans, G. M. Addison, M. K. Palmer, J. H. Scarffe


*Br. J. Cancer* (1980) **41**, 317

***Short Communication***

### LOW IgG OR IgA: A FURTHER INDICATOR OF POOR PROGNOSIS IN CHILDHOOD ACUTE LYMPHOBLASTIC LEUKAEMIA

I. M. HANN*, P. H. MORRIS JONES*, D. I. K. EVANS†, G. M. ADDISON‡, M. K. PALMER§ AND J. H. SCARFFE||

*From the Departments of *Child Health, †Haematology, and ‡Chemical Pathology, Royal Manchester Children's Hospital, Manchester M27 1HA, and the Departments of §Medical Statistics and ||Medical Oncology, Christie Hospital and Holt Radium Institute, Manchester M20 9BX*

Received 20 July 1979 Accepted 9 October 1979

IMMUNOGLOBULIN estimations at diagnosis have been reported in only one large series of children with acute lymphoblastic leukaemia (ALL) (Khalifa *et al.*, 1974). We therefore looked at the initial Ig levels in a large unselected series of children with ALL presenting to the Royal Manchester Children's Hospital Regional Paediatric Oncology Unit between 1971 and 1977. An attempt was made to relate the levels to prognosis, clinical features and response to treatment.

196 consecutive children were investigated before starting treatment. Diagnosis of ALL was made as described by Hayhoe *et al.* (1964). All samples were collected before any blood transfusion was given, stored at $-20°C$ and tested within a week. IgG, IgA and IgM levels were measured using immunodiffusion plates (Hoechst). Low levels of IgA were checked using "low level" plates. Low IgGs were repeated with a less diluted serum. The precision of the assays was 6·6%, 5·8% and 5·6% (coefficient of variation, $n = 12$) for IgG, IgA and IgM respectively. The age-related reference values used were supplied by Hoechst, and are based on several studies reported in the literature (*e.g.* Allansmith *et al.*, 1968) and standardized by relating them to the normal adult level.

155 children (79%) showed Ig levels within the normal range for their age. 31 (16%) had raised levels and 10 (5%) had at least 1 low Ig. 4 had low IgG (Nos. 1, 4, 8 and 9) 4 had low IgA (Nos. 2, 3, 6 and 7) 1 had both low IgG and IgA (No. 5) and 1 had low IgA with high IgM (No. 10). No patient had a low IgM (see Table). None of these children with low Igs had proteinuria, and all had normal blood-albumin levels. The Philadelphia chromosome was not demonstrated in any of the low-immunoglobulin group.

The length of first remission was plotted using the life-table method (Fig.) and differences were analysed for significance using the logrank tests of Peto *et al.* (1977). There was no significant difference between patients with normal and raised Ig. There was, however, a significant deleterious effect of low Ig on remission duration ($P = 0·01$) which was retained even after simultaneous adjustment by the regression method of Cox (1972) for the effects of other known prognostic features (*i.e.* total white-cell count, immunological surface markers, age, mediastinal mass and degree of hepatosplenomegaly).

Studies by Freireich *et al.* (1975) and Hersh *et al.* (1971) at the M. D. Anderson Hospital, Houston, U.S.A., showed that

Correspondence to Dr I. M. Hann, Department of Haematology, Hospital for Sick Children, Great Ormond Street, London WC1N 3JH.

TABLE.—*Low immunoglobulins at diagnosis in childhood lymphoblastic leukaemia*

| Patient | Sex | Age (years) | WBC‡ ($10^9$/l) | Immunoglobulins mg/100 ml* IgG | IgA | IgM | CR time† (days) | First remission (mths) | Second remission (mths) | Survival (days) | Comments |
|---|---|---|---|---|---|---|---|---|---|---|---|
| 1 | M | 8 | 12·5 | 280 (40) | 60 (100) | 100 | 24 | 99+ | – | 100+ | Salmonella septicaemia and prolonged carrier. |
| 2 | M | 7½ | 15·8 | 824 | 35 (57) | 114 | 143 | 9 | 1 | 16 | Difficult inductions |
| 3 | F | 11 | 11·8 | 895 | 44 (60) | 77 | 22 | 7 | 3 | 13 | — |
| 4 | M | 4 | 182·0 | 445 (74) | 50 | 90 | 55 | 11 | nil | 12 | Difficult first-remission induction |
| 5 | F | 4 | 38·3 | 335 (56) | 27 (73) | 210 | — | nil | nil | (48 h) | Died with intracranial haemorrhage |
| 6 | M | 6½ | 4·4 | 930 | 38 (72) | 65 | 29 | 19 | nil | 24+ | Non-B non-T cell |
| 7 | M | 7 | 7·0 | 840 | 32 (67) | 50 | — | nil | nil | 6 | B cell |
| 8 | F | 4 | 15·9 | 500 (83) | 72 | 89 | 28 | 26 | 2 | 29 | Non-B non-T cell |
| 9 | M | 3½ | 19·3 | 485 (80) | 48 | 50 | 27 | 23 | nil | 26+ | Non-B non-T cell |
| 10 | M | 6 | 87·0 | 1500 | 35 (66) | 194 | 23 | 5 | 7 | 19 | — |

‡ At presentation.
† Time from diagnostic marrow to complete remission marrow.
* Bracketed numbers below the relevant Ig level are the percentages of the lower limit of normal.
Only Patient 1 had undue susceptibility to infection, and he is also the only one who has done well. All other patients had strong resistance to chemotherapy, despite other good-risk features in Patients 2, 3, 6, 8 and 9.

FIG.—Length of first remission in children with ALL, according to Ig levels. The "Low" group, with one or more low Ig results, did significantly worse ($P = 0{\cdot}01$) than the other patients. There is no difference between patients with high and normal Ig levels.

cellular immunity of patients with acute leukaemia at diagnosis, and after induction of remission, was related to the subsequent response to treatment. The delayed-hypersensitivity skin test with dermatophyton was the single characteristic significantly related to response. Khalifa *et al.* (1974) studied 120 children with acute leukaemia and demonstrated that those with low IgG at diagnosis had a poor prognosis. We have confirmed this, and also shown that a low IgA may forecast a poor result. Length of first remission was chosen as the main prognostic indicator because of the known very poor survival after relapse of childhood ALL shown by Cornbleet & Chessells (1978).

Membrane surface markers were not available when 6 of our patients presented. Of the other 4, 1 had a B-cell leukaemia, and the other 3 were of the non-B, non-T type. Three patients had an initial white-cell count over $20 \times 10^9$/l, a feature which may be associated with leukaemia of T-cell origin, but no patient had an anterior mediastinal mass. The group is too small for comment on susceptibility to infection, which might be expected to be enhanced, but the one patient who has otherwise done well (Patient 1) had an episode of salmonella septicaemia with subsequent prolonged faecal excretion.

The outstanding feature was the very poor response to treatment, for both induction of remission and re-induction after relapse. Only 1 case (with low initial IgG) responded well. The median lengths of first remission (8 months) and of survival ($17\frac{1}{2}$ months) are also poor. Only 3 of the 10 patients are still alive. All but Patient 1 have relapsed, although 5 had what are considered better risk features (*i.e.* lack of mediastinal mass, initial white-cell count less than $20 \times 10^9$/l and age below 12 years). Two patients (Nos. 2 and 4) needed prolonged treatment before a first remission was induced. Second remissions in 9 of the children were very difficult to achieve. The patient with B-cell leukaemia did not achieve a remission, a feature which has been well described in this type of disease by Freireich *et al.* (1975) and others.

Recent reports from Broder *et al.* (1977, 1978) have shown that some patients with leukaemia of T-cell origin have hypogammaglobulinaemia because the blast cells have suppressor-cell activity. Helper cells have been described in some of Broder's patients with Sezary's syndrome, and both helper and suppressor cells in a T-cell-derived chronic lymphocytic leukaemia patient of Saxon *et al.* (1979). Such findings indicate that neoplastic cells can retain normal function, and may thus provide a valuable supply of human helper and suppressor cells for research. Their characterization may also define patients with acute leukaemia who have exceptionally poor anti-tumour and anti-microbial defences. It would also appear that patients with B-cell and non-B-, non-T (common) ALL may also have a low Ig level which is related to a particularly poor prognosis, and outweighs other factors which would otherwise indicate a good risk. Consequently, identification of patients with low IgA or IgG will allow more accurate stratification of trials for analysis and make possible the selection of these patients for non-conventional intensive treatments such as marrow transplantation.

We are grateful to Mr Trevor Carr for surface marker tests and to Miss E. M. Hammond for immunoglobulin estimations.

### REFERENCES

ALLANSMITH, M., MCLELLAN, B. H., BUTTERWORTH, M. & MALONEY, J. R. (1968) The development of immunoglobulin levels in man. *J. Pediat.*, **72**, 276.

BRODER, S., LAWRENCE, E. & DURM, M. (1977) *Regulatory Mechanisms in Lymphocyte Activation*, Ed. Lucas. New York: Academic Press. p. 689.

BRODER, S., POPLACK, D., WHANG-PENG, J., GOLDMAN, C., MAUL, L. & WALDMANN, T. A. (1978) Characterisation of a suppressor-cell leukaemia. *New Engl. J. Med.*, **298**, 66.

CORNBLEET, M. A. & CHESSELLS, J. M. (1978) Bone marrow relapse in acute lymphoblastic leukaemia in childhood. *Br. Med. J.*, **2**, 104.

COX, D. R. (1972) Regression models and life tables. *J. R. Stat. Soc.* (*B*), **34**, 187.

FREIREICH, E. J., GEHAN, E. A. & BODEY, G. P. (1975) New prognostic factors affecting response and survival in adult acute leukaemia. *Trans. Assoc. Am. Physicians*, **87**, 298.

HAYHOE, F. G., QUAGLINO, D. & DOLL, R. (1964) The cytology and cytochemistry of acute leukaemias. *M.R.C. Special Report*, Series. London: HMSO. No. 304.

HERSH, E. M., WHITECAR, J. P., MCCREDIE, K. B., BODEY, G. P. & FREIREICH, E. J. (1971) Chemotherapy, immunocompetence and prognosis in acute leukaemia. *New Engl. J. Med.*, **285**, 1211.

KHALIFA, A. S., TAKE, H., CEJKA, J. & ZUELZER, W. W. (1974) Immunoglobulins in acute leukaemia in children. *J. Pediat.*, **85**, 788.

PETO, R., PIKE, P. C., ARMITAGE, P. & 7 others (1977). Design and analysis of randomized clinical trials requiring prolonged observation of each patient. II. Analysis and examples. *Br. J. Cancer*, **35**, 1.

SAXON, A., STEVENS, R. H. & GOLDE, D. W. (1979) Helper and suppressor T-lymphocyte leukaemia in ataxia-telangiectasia. *New Engl. J. Med.*, **300**, 600.